# Mendelian randomization study on the causal effects of systemic lupus erythematosus on major depressive disorder

**DOI:** 10.1038/s10038-022-01080-7

**Published:** 2022-11-01

**Authors:** Wenchang Li, Hoktim Kan, Weizhe Zhang, Yanlin Zhong, Weiming Liao, Guiwu Huang, Peihui Wu

**Affiliations:** 1 Department of Joint Surgery, The First Affiliated Hospital of Sun Yat-sen University, Sun Yat-sen University, Guangzhou, China; 2 Guangdong Provincial Key Laboratory of Orthopedics and Traumatology, The First Affiliated Hospital of Sun Yat-sen University, Sun Yat-sen University, Guangzhou, China; 3 Sun Yat-sen Memorial Hospital, Sun Yat-sen University, Guangzhou, China

**Keywords:** Genome-wide association studies, Depression, Systemic lupus erythematosus

## Abstract

The vast majority of epidemiological studies suggested a link between systemic lupus erythematosus (SLE) and major depressive disorder (MDD). However, the causality for SLE on the risk of MDD remained unknown due to confounding factors or reverse causality. Herein, we investigated the causality between SLE and MDD in those of European ancestry by a Mendelian randomization (MR) approach. Summary genetic data of cases with SLE/MDD were derived from independent largest public genome-wide association study. Forty-six single nucleotide polymorphisms associated with SLE were used as instrumental variables. The main causal inference was carried out using the MRE-IVW method. Additional, reverse-direction MR and multivariable MR analyses were further performed. Result indicated that SLE was causally associated with a lower risk of MDD (using the MRE-IVW method, odds ratio [OR] = 0.983, 95% confidence interval [CI] = 0.974–0.991, *p* = 1.18 × 10^−4^). Complementary analysis found no heterogeneity or horizontal pleiotropy. Multivariate MR analysis yielded consistent results (OR =  0.981; 95% CI = 0.969–0.993; *p* = 2.75 × 10^−3^). Reverse-direction MR analysis suggested non-causal relationship of MDD on the risk of SLE (using the IVW method, OR = 0.846, 95% CI = 0.345–2.072; *p* = 0.714). Thus, this is the first study providing evidence of potential causal links between SLE and MDD and further related research is needed.

## Introduction

Major depressive disorder (MDD) is one of the most severe and common disorders in psychiatry globally and has long been a major societal concern [1]. MDD affects more than 300 million people of all ages worldwide, and is currently a major contributor to the global disease burden in the general population [2, 3]. However, the pathogenesis of MDD is unclear. Twin studies have shown that 30–40% of the variation in MDD can be attributed to genetic factors [4]. Notably, MDD has long been regarded as a “comorbidity” of several chronic diseases, such as angina, systemic lupus erythematosus (SLE), arthritis and diabetes, which worsens the quality of health substantially compared with when these diseases occur alone [5].

Psychological disorders in SLE have been investigated in recent decades. The reported prevalence varied widely across several published SLE cross-sectional studies, from 2.1% to 78.6% depending on factors such as study design and diagnostic criteria [6–9]. In the vast majority of epidemiological reports, the prevalence of depression in SLE patients was approximately twice that in the general healthy population in clinical and community samples [10]. SLE accompanied by depression is associated with markedly worse prognosis in physical, mental, and social domains. Given this very close relationship between SLE and MDD, diagnosing and treating MDD may help improve health-related quality of life in individuals with SLE [11]. However, research and evaluation from observational studies are insufficient to draw conclusions on the cause–effect relationships due to spurious correlations caused by confounders and reverse causality.

Well-designed randomized controlled trials (RCTs)—the gold standard to imply causality—can tackle the potential confounders effectively. However, RCTs take considerable time and might be impractical to initiate due to ethical concerns and financial limitations. As an important complementary causal research approach, Mendelian randomization (MR) uses genetic variants that associate with the exposure as instrumental variables to robustly assess the causality between exposure and outcome, given that certain assumptions including the absence of pleiotropy are met [12]. Against this background, the purpose of this study is to investigate lifetime prevalence rates of MDD in patients with SLE, which extends previous work by simultaneously assessing the largest GWAS data of MDD in a large sample of SLE patients, using a reliable and validated structured MR approach.

## Methods

### GWAS data sources

This two-sample MR study using publicly available summary statistics of GWAS data on SLE [13] and MDD [14]. SLE-related instrumental variables were derived from independent genome-wide relationship studies (GWAS), including 7219 cases and 15,991 controls with European ancestry. Genetic relationships with MDD were obtained from the GWAS data among individuals of European ancestry from the Psychiatric Genomics Consortium database, which comprises 135,458 major depression cases and 344,901 controls. Among them, 59,851 patients with MDD and 113,154 controls were included in the present MR analysis, because genome-wide summary statistics of 23andMe data were not public available (75,607 cases and 231,747 controls). Further details concerning the above studies have been published previously [13, 14].

### Genetic instrument selection process

SNPs are considered to meet the following three key assumptions [15]. (1) Genetic variants should be strongly associated with the exposure. The selection of instrumental variables should satisfy the association between SNPs and the corresponding phenotype (systemic lupus erythematosus) (*p* < 5 × 10^−8^). (2) Genetic variants extracted for exposure should be independent of any confounder. (3) The genetic variants only affect the outcome only through the exposure. In order to meet the following assumptions, SNPs are then filtered through the following steps. Candidate genetic instrumental variables (IVs) that surpassed a conventional genome-wide significance threshold (*p* < 5  ×  10^−8^) were obtained from a recent GWAS of SLE comprising data on participants with European ancestry. Proxy SNPs were identified at a cut-off of R^2^ > 0.8 to replace missing SNPs in the outcome GWAS dataset. If no suitable proxy was available, SNPs were discarded. Linkage disequilibrium (LD) clumping with a clumping window of 10 MB was applied to ensure that these SNPs were individually, and cumulatively, considered as valid instruments for MR analysis (LD R^2^ > 0.01) [16]. F-statistic was used to confirm the strength of IVs, with weak IVs (F-statistic <10) being discarded. In the harmonization process, ambiguous and palindromic SNPs (minor allele frequency >0.42) were excluded. Outlier SNPs with potential pleiotropy was detected by the MR-pleiotropy residual sum and outlier (MR-PRESSO) test and then discard.

### Two-sample Mendelian randomization

To perform a robust and reliable inference of the causal relationship between SLE and MDD, in the main analysis, we performed multiplicative random-effect inverse variance weighted (MRE-IVW) analysis [17]. MR-Egger regression and weighted median constitute statistical tests for the presence of pleiotropic effects of SNPs under analysis and provide a complementary causal estimate [18, 19]. The Cochran Q test for the IVW method was implemented to detect heterogeneity [20]. In detail, no heterogeneity was detected if the *p* value of the Cochran Q was >0.05 and *I*^*2*^ was <25%. The leave-one-out test was then performed to assess whether the IVW estimate was biased by the influence of particular single SNPs. Additionally, reverse-direction MR analysis was conducted to examine whether there existed reverse-direction causal relationship. Statistical analyses were performed using R software version 4.0.2 (https://www.r-project.org/) with the two-sample MR package (version 0.5.5).

In addition, each SNP was looked up in the genetic instrument in Phenoscanner (http://www.phenoscanner.medschl.cam.ac.uk/) to determine whether the estimate was violated by potential risk factors verified by other MR studies, including periodontitis [21], plasma cathepsin B level [22], gut microbiome [23], selenium [24], circulating GDF-15 level [25], and high serum iron status [26].

### Power calculation

An online publicly available power calculator (mRnd, http://cnsgenomics.com/shiny/mRnd/) was utilized to evaluate the power of our study [27]. For binary outcomes (MDD), after we inputted the required parameters in mRnd (α = 0.05, R^2^ = 0.983 in this study), the power of our study was roughly estimated.

### Multivariable Mendelian randomization

Taking critical impact of several confounding factors linking SLE to the MDD onset into account, a multivariable MR analysis was applied to estimate the effect of multiple exposure variables on an outcome (MDD in this study). For MVMR analyses, we constructed instruments using SNPs in each of the GWASs meeting our single-variable MR selection criteria, described previously. We combined the SNPs from the relevant GWASs (Body mass index [28], smoking [29], drinking [29] and physical activity [30]) and removed those SNPs which was missed in one or more datasets, then extracted the SNP effects and corresponding standard errors from the exposures and outcome GWASs. Notably, SNPs with robust information related to both causal SLE and four several confounders (see Supplement table S4) were utilized as IVs for multivariable MR analysis. Inverse-variance weighted method was further used to estimate the causal effect.

## Results

### Two-sample Mendelian randomization analysis for causal link of SLE with MDD

**Fig. 1:**
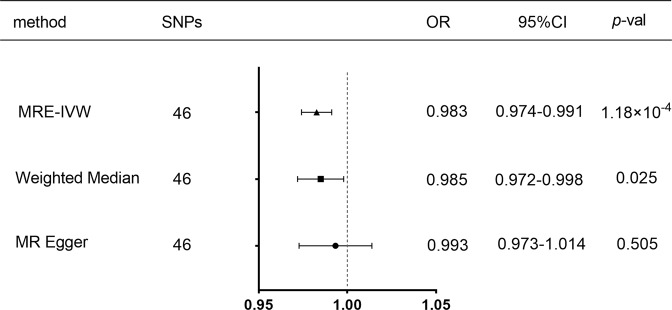
Forest plot of Mendelian randomization analyses for the relevance of systemic lupus erythematosus with risk of major depressive disorder. OR odds ratio, CI confidence interval, MREIVW multiplicative random effects inverse variance weighted method, MR Mendelian randomization, SNP single-nucleotide polymorphism

**Fig. 2:**
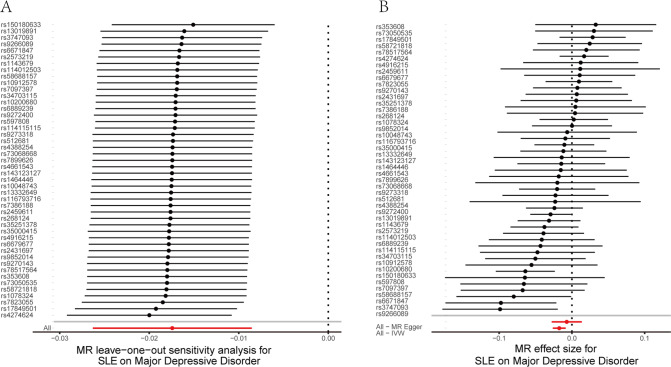
**A** Plots of “leave-one-out” analyses for MR analyses of the causal effect of systemic lupus erythematosus with the risk of major depressive disorder (MDD). **B** Forest plots of instrumental variable Wald ratios and causal effect assesses of the relationship between systemic lupus erythematosus and the risk of MDD observed in the multiplicative random effects inverse variance weighted (MRE-IVW) method-based MR approach. The horizontal lines in the figure represents beta value and its 95% confidence interval [CI] of causal inference, which indicates the genetic effect of the SNP on MDD

After the clumping process, 52 LD-independent SNPs for exposure (SLE) remained for further analysis. Among them, 4 outlying SNP (rs1270942, rs13136219, rs501480, rs7768653) in the causality inference was detected based on MR-PRESSO analysis and excluded. Two palindromic SNPs (rs115531193, rs2736332) were detected and removed in the harmonization process. 46 SNP selected as instrumental variables were listed in Supplementary Table S1. As shown in Fig. 1, the overall causal relationship between SLE and MDD (IVW method, OR = 0.983; 95% CI, 0.974 to 0.991; *p* = 1.18 × 10^−4^) was significant. In addition, results from the “leave-one-out” analysis (Fig. 2A) demonstrated that no single SNP was driving the IVW point estimate. These results indicated that SLE was negatively associated with the risk of MDD. Figure 2B showed the forest plot of pooled MR estimates and individual estimates between SLE-associated IV and the risk for MDD. Finally, conducting reverse MR analysis with available SNPs listed in Supplementary Table S2, we gave the evidence that there is not causal effect of MDD on the risk of SLE (IVW method, OR = 0.846; 95% CI, 0.345 to 2.072; *p* = 0.714). However, we had limited power (27%) to test significant causal effect of SLE on the risk of MDD, possibly due to small sample size of the MDD GWAS and the ORs for the relationship was relatively limited.

### Sensitivity analysis

**Fig. 3:**
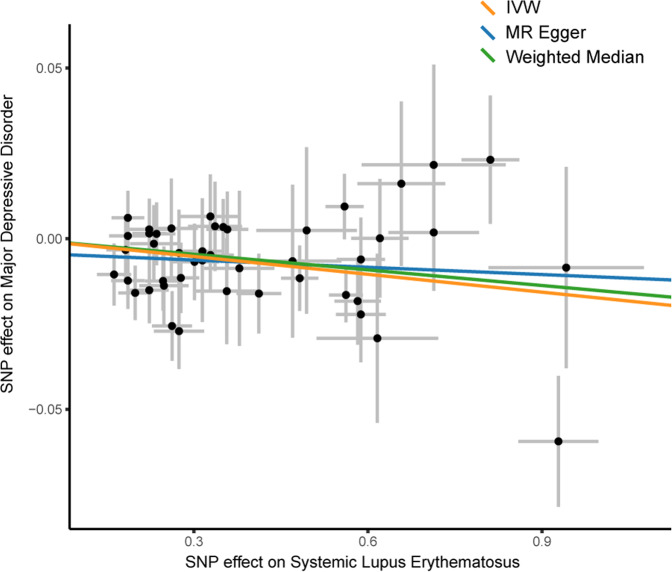
Single-nucleotide polymorphism (SNP) exposure relationship estimates for systemic lupus erythematosus (SLE) against the SNP-outcome relationship estimates for major depressive disorder (MDD). Causal effect given by each Mendelian randomization (MR) estimator, caveated by issues discussed in the main text. MRE-IVW multiplicative random effects inverse variance weighted method, SNP singlenucleotide polymorphism. The X-axis and Y-axis scale represent the beta value (genetic effect) of the SNP on the risk of SLE and MDD, respectively

**Fig. 4:**
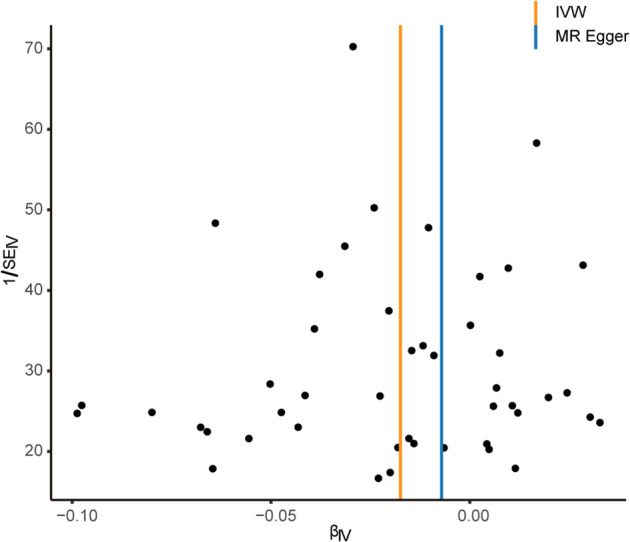
Funnel plot showing the inverse variance weighted MR estimate of each systemic lupus erythematosus SNP with estimated major depressive disorder versus 1/standard error (1/SEIV)

Assessment of sensitivity analysis scores based on IVW analysis were consistent with weighted median and MR-Egger results. Figure 3 shows the scatter plot of the causal effect given by each MR estimator. The MR-Egger regression revealed that directional pleiotropy was unlikely to bias the result (Egger_intercept = −0.004, *p* = 0.283). Cochran Q test and the funnel test (Fig. 4) indicated no heterogeneity between SLE and MDD (Q value = 43.306, *p* = 0.544). And the result of the weighted median further supported the positive relationship, which confirmed that the results were not biased by heterogeneity. Moreover, our results of *I*^*2*^ value showed the absence of heterogeneity (*I*^*2*^ = 0%), indicating increased reliability of MR estimates. The Phenoscanner results of each SNP with the genetic traits are shown in Supplementary Table S3. No potential risk factors were detected to violate the robustness of our MR causality estimate.

### Multivariable Mendelian randomization

Using a threshold of *p* < 5 × 10^–8^, those IVs after quality control were utilized to estimate the causal effect in MVMR were listed in Supplement table S4. There was strong evidence that SLE was causally associated with a lower risk of MDD, and with MVMR after conditioning with other four traits, the causal relationships was still robust (IVW method, OR = 0.981; 95% CI, 0.969 to 0.993; *p* = 2.75 × 10^−3^). Smoking and BMI was also causally associated with the risk of MDD (IVW method, BMI: OR = 1.085; 95% CI, 1.016 to 1.159; *p* = 0.016; Smoking: OR = 1.468; 95% CI, 1.236 to 1.744; *p* = 1.23 × 10^−5^). However, drinking and physical activity were detected insignificant causal effect on the risk of MDD (*p* value for drinking is 0.514 and for physical activity is 0.056). In a conclusion, known from the result of MVMR, the causal relationship between SLE and MDD was robust and it wouldn’t be biaed by these confounding factors.

## Discussion

This study obtained partly genetic evidence in support of the potential causal links between SLE and the lower risk of MDD by applying a validated structured MR approach. This relationship was significant in the main MR analyses and consistent across follow-up sensitivity analyses. These findings demonstrated that SLE patients tended to have a lower prevalence of MDD in genetics, which might be contrary to previous observational studies.

Observational studies have reported inconsistent findings on the relationship between SLE and MDD. That MDD was a risk factor on SLE disease activity have been reported in some cohorts, but in other cohorts, MDD prevalence was independent to SLE disease activity [31–35]. Study of Roberts et al. suggested that MDD increases the risk of SLE [36]. However, another study demonstrated that improving patients’ mood did not significantly ameliorate the disease activity of SLE [37]. Previous studies reported that serum anti-ribosomal P (anti-RP) titers were significantly more likely to be positive in SLE with MDD than without, implying that anti-RP plays a role in SLE-mediated depression [38]. In addition, the regulatory relationship of SLE on depression may also be related to neuroinflammation and brain serotonin levels [39, 40]. Huang et al. analyzed data from a cohort of 1609 SLE patients who had no history of MDD prior and made a multivariate analysis, suggested that glucocorticoid use and skin manifestations were predictors of depression, but global disease activity of SLE was not. Interestingly, the authors found that the incidence of depression decreased as the time to SLE diagnosis increased, which may be due to better control of disease activity, less prednisone used and coping ability increased over time [41]. Stojan et al. reported that 59% of SLE patients experienced a significant decrease in BMI within 5 years [42]. Our study demonstrated that smoking and BMI are clear predictors of MDD and the relationship between lower BMI and lower risk of MDD was verified in our multivariable MR. Undoubtedly, it is necessary to explore the potential causal relationship of MDD to SLE at the gene level.

Some limitations could potentially bias the results of observational studies. Firstly, these observational studies cannot be used as direct evidence of the causal relationship between SLE and depression because of its design. Secondly, most studies used questionnaire reports to define depression, which may deviate from the strict definition of “major depression disorder”. Thirdly, most original reports lacked the assessment of attribution to MDD and fail to exclude confounding factors (such as drugs, smoking, BMI, etc.). Mental and physical health are tightly connected. When depressive symptom coexists with the development of SLE, health-related quality of life, disability, and costs tend to be much worse [11, 43, 44]. The relationship between MDD and SLE may be related to social income and compliance [45–47].

MR studies use genetic variation as a statistical tool and has been widely used for evaluating causal inference between disease risk factors and exposure outcomes. Our results showed that SLE was associated with the lower risk of MDD and MDD had no significant causal relationship with SLE. To date, this is the first MR study to explore the causal relationship between SLE and MDD. In this two-sample MR study, the potential causal relationship between genetically predicted SLE and MDD was investigated thoroughly. Instrumental variables were chosen from corresponding largest summary statistics of GWAS datasets after a set of rigorous process. Moreover, the absence of pleiotropic and heterogeneity minimized the effects of confounded estimates caused by single SNPs that could affect the outcome on different pathways. In addition, ancestry was controlled by selecting European samples in this MR study may help to minimize bias of the unmatched genetic variants frequencies among different ancestry. This MR analysis showed that SLE may have a mild protective causal relationship with MDD. This contrasts sharply with previous observational studies, thus, the mechanism of the potential protective effect of SLE on MDD needs further exploration.

Several limitations also exit in this study. First, only Europeans ancestry were included, and additional studies should be conducted to confirm whether our findings are generalizable in ethnically. Second, although we have performed multivariate MR analysis for possible potential confounders such as BMI, smoking, drinking, physical activity, we did not obtain gender or drugs information because of using summary data, so the impact of sex hormone or drugs differences on the results cannot be excluded. Although our results are contrary to previous observational studies, it shows that the relationship between SLE and MDD is still very complex, which needs further rigorous disease diagnosis and more detailed classification research.

## Supplementary information

Supplemental Materials

## Data Availability

The original contributions presented in the study are included in the article/Supplementary Material, further inquiries can be directed to the corresponding author.
